# A Quasi-direct LC-MS/MS-based Targeted Proteomics Approach for miRNA Quantification *via* a Covalently Immobilized DNA-peptide Probe

**DOI:** 10.1038/s41598-017-05495-7

**Published:** 2017-07-18

**Authors:** Liang Liu, Qingqing Xu, Shuai Hao, Yun Chen

**Affiliations:** School of Pharmacy, Nanjing Medical University, Nanjing, 211166 China

## Abstract

MicroRNAs (miRNAs) play a vital role in regulating gene expression and are associated with a variety of cancers, including breast cancer. Their distorted and unique expression is a potential marker in clinical diagnoses and prognoses. Thus, accurate determination of miRNA expression levels is a prerequisite for their applications. However, the assays currently available for miRNA detection typically require pre-enrichment, amplification and labeling steps, and most of the assays are only semi-quantitative. Therefore, we developed a quasi-direct liquid chromatography-tandem mass spectrometry (LC-MS/MS)-based targeted proteomics approach to quantify target miRNA by innovatively converting the miRNA signal into the mass response of a reporter peptide *via* a covalently immobilized DNA-peptide probe. Specifically, the probe containing the targeted proteomics-selected substrate/reporter peptide, GDRAVQLGVDPFR/AVQLGVDPFR, and the DNA sequence complementary to the target miRNA (i.e., miR-21) was first immobilized on APMTS modified silica nanoparticles using PDITC. After the immobilized probe was recognized and hybridized with the target miRNA, the excess probe was degraded using MBN and followed by a trypsin digestion of the hybrids. The reporter peptide was released and quantified using LC-MS/MS. The obtained LOQ was 5 pM. Finally, the developed assay was used for the quantitative analysis of miR-21 in breast cells and tissue samples.

## Introduction

MicroRNAs (miRNAs) are single-stranded, noncoding RNAs with a typical length of 18–25 nucleotides. They generally take part in a number of biological processes as regulatory factors *via* repression of messenger RNA (mRNA) translation^1–3^. Recently, accumulating evidence has demonstrated that miRNA plays a very important role in the development of various cancers, including breast cancer^4–7^. For example, the differential levels of miRNA in healthy volunteers and breast cancer patients indicated the potential use of miRNA as a diagnostic/prognostic marker and therapeutic target^5, 8, 9^. Therefore, accurate determination of miRNA expression levels is a prerequisite for their application.

The relatively short length and low abundance of miRNAs are disadvantages that must be overcome in a detection platform^10, 11^. To date, many detection techniques have been reported for the study of the miRNA expression profile, including indirect methods (e.g., microarrays^12^, polymerase chain reaction (PCR)^13, 14^, next-generation sequencing^15^, electrocatalysis^16^ and fluorescent resonance energy transfer (FRET))^17^ and direct methods (e.g., differential interference contrast (DIC) imaging^18^, spectral detection assisted by duplex-specific nuclease^10^, electrochemical-based methods and capillary electrophoresis (CE)-based methods)^19, 20^. Indirect detection methods normally require pre-amplification and chemical or enzymatic modification of the target miRNA. These additional steps often result in information loss, increased assay time and sequence-related biases during quantification^20^. Notably, most of these methods made use of fluorescence detection. In a previous study, the beads array for the detection of gene expression (BADGE) assay immobilized oligonucleotide capture probes on fluorescence microspheres, and the beads were then hybridized to labeled cRNA and passed through a counting device, which records the identity of each microsphere and its probe intensity^21^. The subsequent studies mostly combined amplification with fluorescence detection to increase the sensitivity, e.g., using PCR^22^ or some enzyme-aided target recycling techniques^23^. However, fluorescence signals are easily affected by temperature and pH^24^. In addition, spectral separation often requires a difference in fluorescence for multiplex analysis, which could be difficult^25^. Direct detection techniques are promising, but they are primarily in the early stages of development and still require significant efforts to make them applicable. Furthermore, most assays only provide a limited degree of qualitative data^26^. Thus, cross comparison could be difficult among different studies (or different laboratories)^27^. With the recent increasing demand for the detection of slight deregulations in miRNA levels rather than the presence or absence of a particular miRNA species, techniques that provide high quantitative accuracy are of great interest. Consequently, a mass spectrometry technique, liquid chromatography-tandem mass spectrometry (LC-MS/MS)-based targeted proteomics, has attracted our attention.

LC-MS/MS-based targeted proteomics has become an indispensable tool for protein quantification in recent years^28–30^, mainly because of the remarkable performance of modern mass spectrometry (e.g., high sensitivity, high selectivity and wide dynamic range)^31, 32^. The key concept of this technique is to specifically determine the protein of interest at the peptide level^33^. Peptides generated from the proteolytic digestion of the target protein serve as surrogate analytes. Selected or multiple reaction monitoring (SRM or MRM) can be used to quantify the selected surrogate peptides^30^. However, the application of mass spectrometry to directly detect miRNA has encountered some difficulties due to the complicated and unresolved mass spectra of miRNA, which was discussed in previous studies^34–37^. Within this context, we introduced the concept of a surrogate peptide into miRNA quantification and developed a targeted proteomics approach^38^. Specifically, the target miRNA was biotinylated and attached to streptavidin agarose in advance. Its signal was then converted into a reporter peptide *via* a self-designed DNA-peptide probe, and the reporter peptide was ultimately quantified using LC-MS/MS. Similar to other indirect methods, the target miRNA modification is susceptible to several issues. In addition to the previously mentioned issues, the biotinylation kit uses T4 RNA ligase to conjugate a single nucleotide analog to the 3′ terminus of the RNA strand^39^. This conjugation reaction is not very efficient (~70%), and both intramolecular and intermolecular joining of RNA molecules are possible^40, 41^. Second, even though biotin and streptavidin form one of the strongest non-covalent interactions (with a dissociation constant of ~10^−15^ M)^42^, it is still not a covalent bond. Dissociation is possible in response to a change in the ionic strength and/or temperature^43^. Third, the ubiquitous presence of biotin in eukaryotic cells could have a significant interfering impact on the miRNA loading efficiency^44^.

**Figure 1:**
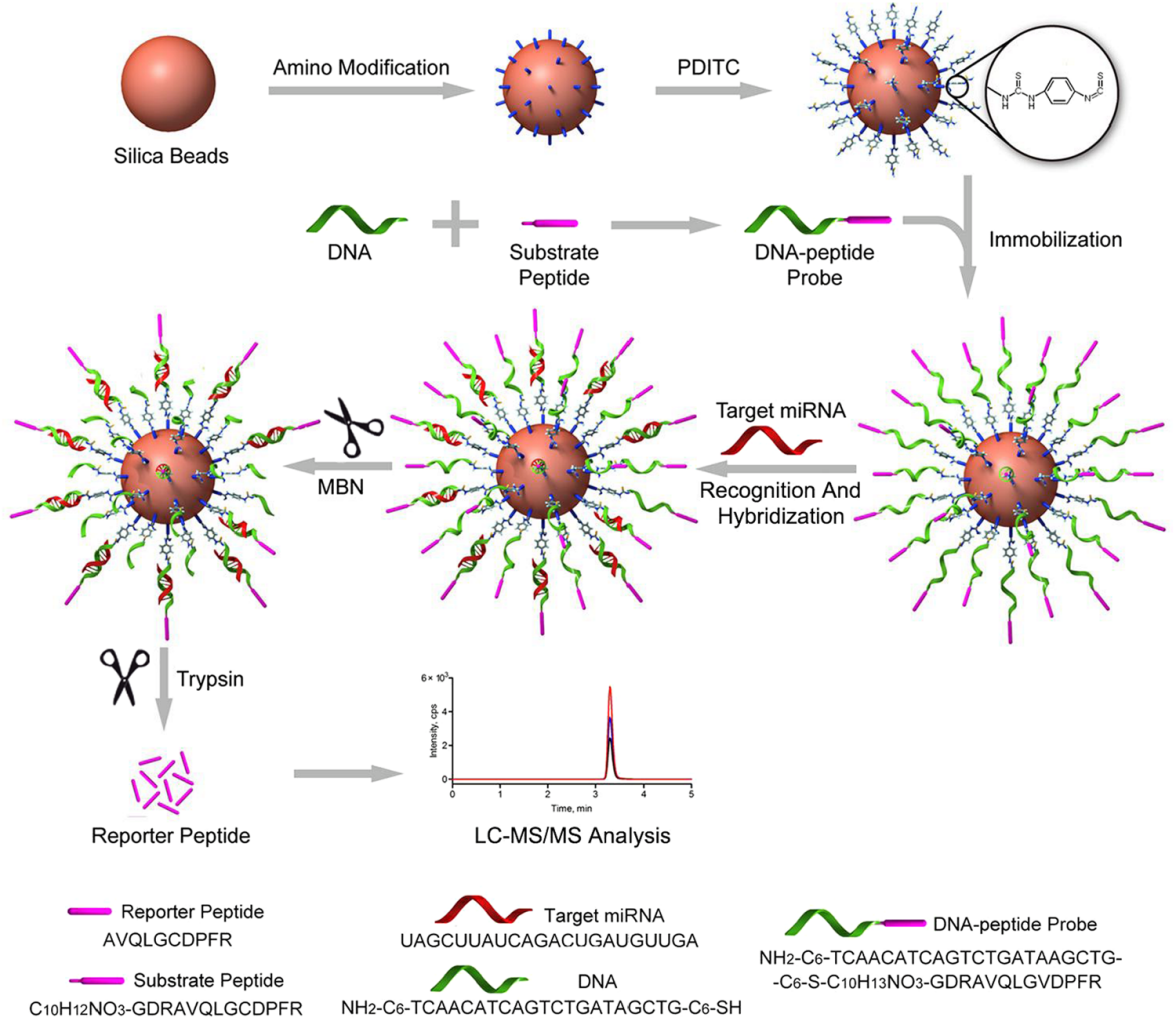
Schematic representation of quasi-direct targeted proteomics approach for miRNA quantification. Substrate peptide containing the sequence of reporter peptide and tryptic cleavage site was tagged to DNA at 3′ end, which was complementary to the target miRNA. With PDITC reaction, the DNA-peptide probe was immobilized on the amino-modified silica nanoparticles. This immobilized probe was then hybridized with the target miRNA, followed by sequential digestion by mung bean nuclease and trypsin. The reporter peptide was ultimately released and quantified using LC-MS/MS. In this way, the quantity of target miRNA can be inferred from the measurement of reporter peptide.

In this study, we developed a quasi-direct targeted proteomics approach by covalently immobilizing a DNA-peptide probe on amino-modified silica nanoparticles (Fig. 1). A substrate peptide containing the reporter peptide and tryptic cleavage site was first designed using a targeted proteomics rationale and then conjugated with a DNA sequence complementary to the target miRNA. After the *p*-phenylene diisothiocyanate (PDITC) reaction, the newly formed DNA-peptide probe was immobilized on silica beads that were amino modified using (3-aminopropyl) trimethoxysilane (APTMS) in advance. This immobilized probe was then hybridized with the target miRNA (i.e., miR-21). After removing the excess probe using mung bean nuclease (MBN), the reporter peptide was liberated from the hybrids using trypsin and ultimately quantified using LC-MS/MS. The new assay for miR-21 was optimized for the parameters including conjugation, immobilization, hybridization and digestion. Finally, miR-21 was quantified in 3 breast cancer cell lines and 36 pairs of human breast primary tumors and adjacent normal tissue samples. The obtained data were compared with the quantitative reverse transcription PCR (qRT-PCR) results.

## Materials and Methods

### Chemicals and Reagents

Maleimidohexanoic acid-modified substrate peptide (Male-GDRAVQLGVDPF -R), reporter peptide (AVQLGVDPFR) as well as internal standard peptide containing stable-isotope labeled amino acids were synthesize by ChinaPeptides Co., Ltd. (Shanghai, China). Purification of the peptides was also provided by the manufacturer. The stable isotope-labeled amino acid was supplied by Cambridge Isotope Laboratories, Inc. (Andover, USA). miR-21 with/without mismatches, and its complementary DNA both with a disulfide modification at 5′ end and amino modification at 3′ end were custom synthesized by Genscript (Nanjing, China). miR-21 has the following sequence, 5′-UAGCUUAUCAGACUGAUGUUGA-3′. The corresponding DNA is 5′-amino-C_6_-TCAACATCAGTCTGATAAGCTA-C_6_-thiol-3′. PDITC was provided by Acros Organics (Geel, Belgium). Silica nanoparticles (40 nm) were supplied by Haotian technology Co., Ltd. (Shanghai, China). Ammonium bicarbonate (NH_4_HCO_3_) was obtained from Qiangshun Chemical Reagent Co., Ltd. (Shanghai, China). Immobilized TCEP reducing beads were obtained from Thermo Scientific (Rockford, USA). Sequencing grade modified trypsin and MBN with its reaction buffer were both purchased from Promega (Madison, USA). Phosphate buffered saline (PBS) was purchased from Beyotime Institute of Biotechnology (Nantong, China). Acetonitrile (ACN) and methanol were obtained from Tedia Company, Inc. (Fairfield, USA). Cyanotic chloride, APTMS and trifluoroacetic acid (TFA) were gained from Aladdin Chemistry Co., Ltd. (Shanghai, China). Diisopropyl ethylamine (DIEA), dimethylformamide (DMF) and 9-fluorenylmethyl chloroformate (Fmoc-Cl) were both purchased from Sinopharm Chemical Reagent Co., Ltd. (Shanghai, China). Formic acid (FA) and sodium borate were obtained from Xilong Chemical Co., Ltd. (Shantou, China). Dulbecco’s Modified Eagle Media (DMEM), fetal bovine serum and penicillin/streptomycin solutions were obtained from Thermo Scientific HyClone (Logan, USA). Trypan blue and sodium dodecyl sulfate (SDS) were obtained from Generay Biotech Co., Ltd (Shanghai, China). All the solutions used in the experiments were prepared in DEPC-treated water (Beyotime Institute of Biotechnology, Nantong, China).

### Preparation of Stock Solutions, Calibration Standards and Quality Controls (QCs)

A 100 μM stock solution of miR-21 in DEPC water was prepared and stored in an amber glass tube to protect it from light at −20 °C. The calibration standards were created by serially diluting the stock solutions in a 10 μM tRNA library from baker’s yeast (Sigma, St. Louis, MO, USA)^45^. The concentrations of the calibration standards were 5 pM, 10 pM, 25 pM, 100 pM, 1 nM, and 10 nM. The QC standards for the lower limit of quantification (LLOQ), low QC, mid QC and high QC were prepared at 5 pM, 15 pM, 500 pM and 8 nM and frozen prior to use. For the reporter peptide, the corresponding isotope-labeled synthetic peptide was used as the internal standard. A 100 μM internal standard stock solution was prepared, and a 1 nM working solution was prepared by diluting the stock solution with an ACN:water mixture (50:50, *v*/*v*) containing 0.1% FA.

### Cell Culture and Tissue Collection

MCF-10A cells (ATCC, Manassas, VA) and MCF-7/WT (ATCC, Manassas, VA) cells were cultured routinely in DMEM media supplemented with 10% fetal bovine serum and 1% penicillin/streptomycin in a humidified atmosphere with 5% CO_2_ at 37 °C. MCF-7/ADR (Keygen Biotech, Nanjing, China) cells were grown in an RPMI 1640 media (with L-glutamine and sodium pyruvate) supplemented with 10% fetal bovine serum and 1% penicillin/streptomycin at 37 °C under a 5% CO_2_ atmosphere. Cells were split every 5–7 days by lifting the cells with 0.25% trypsin, and the cells were maintained by the addition of fresh medium. The MCF-7/ADR cells were periodically reselected by growing them with 1000 ng/mL DOX to maintain a highly drug-resistant cell population^46^. Experiments were conducted using the cells incubated without DOX for 48 h. The cells were counted using a hemocytometer (Qiujing, Shanghai, China). The cell viability was assessed using trypan blue (0.4%) exclusion. The cell suspensions, PBS and trypan blue were mixed in a 2:3:5 ratio, and the viable cells were counted after incubation for 5 min at 37 °C.

The breast tissue collection in the present study was approved by the ethical review committee of Nanjing Medical University, and informed consent was obtained from all patients. All experiments were performed in accordance with the relevant guidelines and regulations. Thirty-six pairs of tumors and adjacent normal tissue samples were collected from breast cancer patients between January 2012 and December 2012 at the First Affiliated Hospital of Nanjing Medical University and Nanjing Drum Tower Hospital, Nanjing, China (mean patient age, 52.9 ± 8.6 years; age range, 38–65 years). The pathologic examinations of the tissue sections were confirmed as normal and/or cancerous by the hospital pathologists. The histological evaluations of the adjacent tissue samples indicated that there was no contamination from the tumor or other abnormal cells. All the participants were biologically unrelated and belonged to the Han Chinese ethnic group from the Jiangsu province in China. After collection, the samples were stripped of adhering fat, cut into small pieces, and stored at −80 °C. Before RNA extraction, they were thawed to room temperature and rinsed thoroughly with DEPC water. The total RNA was isolated from the cells, and using TRIzol Reagent, the concentrations of approximately 50 mg tissue homogenates were estimated using a nanodrop spectrophotometer (Thermo Fisher Scientific Inc., MA, USA). The RNA extracts were stored at −80 °C until further processing.

### Preparation of the DNA-peptide Probe

The experimental procedure to prepare the DNA-peptide probe has been described in our previous work^38^. Briefly, 1 OD DNA (4.55 nmol) was first reduced using 300 μL of TCEP reducing beads at 37 °C for 2 h with vigorous shaking. After centrifugation at 1000 g for 5 min, the supernatant was collected, and an equal volume of 50 nmol maleimidohexanoic acid-modified substrate peptide was added to the solution. Prior to purification, the conjugation reaction was performed at 37 °C for 4 h with vigorous shaking. The DNA-peptide compound was separated from the excess non-conjugated DNA and peptide using high-performance liquid chromatography (HPLC). Finally, the collected fraction was ultra-filtered using an Amicon Ultra 3 K device (Merck Millipore, Darmstadt, Germany). Quantification was performed using an external calibration peak area measurement. The HPLC conditions are provided in the Supporting Information.

### Amino Modification of the Silica Beads and Amino Loading Estimation

Silica beads (~50 mg) were washed with ethanol and resuspended in 1 mL of ethanol, followed by an addition of 10 μL (1%) APTMS and shaking for 1 h at room temperature. After amino modification, the beads were washed 5 times with ethanol and resuspended in 1 mL of methylene chloride, which was followed by an addition of 20 μL (0.12 mmol) of DIEA and sonication for 5 min. To estimate the available amino groups, 25.8 mg (0.1 mmol) of Fmoc-Cl were added into the suspension, and the mixture was shaken for 1 h at room temperature. Following centrifugation at 5000 rpm for 5 min, the beads were washed with methylene chloride and ACN 5 times, sequentially. In this study, the unreacted amino groups were capped using a mixture of 0.2 M acetic anhydride and 0.2 M DIEA in methylene chloride (1 mL for 50 mg of beads) with shaking overnight at room temperature. The beads were then washed with methylene chloride and ACN 5 times again. Finally, the Fmoc protecting group was removed using 1 mL of 20% piperidine in DMF. After shaking for 30 min, the supernatant was collected and analyzed using HPLC.

### Immobilization of the DNA-peptide Probe

The amino-modified beads (50 mg) were suspended in 1 mL of DMF containing 10% pyridine and 0.2% PDITC and shaken for 2 h at room temperature. The beads were then washed with DMF 5 times, with ethanol 3 times and with methylene chloride 3 times. Afterward, 0.75 nmol of the DNA-peptide probe and 10 mg of the beads were mixed in 400 μL of the reaction buffer, which included 2 M sodium chloride and 0.05 M sodium borate buffer at pH 8.5. After shaking overnight at 37 °C, the beads were washed with PBS 3 times.

### miRNA Hybridization with the DNA-peptide Probe

After immobilization, 1 nM miR-21 in the hybridization buffer (200 μL) was mixed with 20 μL of the probe immobilized beads (0.25 mg), and the reaction was conducted in an oven. Similar to previous work^38^, the hybridization buffer (Table 1S), time and temperature were optimized. After hybridization, the beads were thoroughly washed to remove any unbound miRNAs. To evaluate the hybridization specificity of the probe, the miR-21 molecules with a single-base mismatch (5′-UAGCUUAUCAGUCUGAUGUUGA-3′) and two-base mismatch (5′-UAGCUUAUCAGUGUGAUGUUGA-3′) sequences were also examined. The underlined bases in the sequences represent the mismatch sites.

### In-solution MBN Digestion and Tryptic Digestion

After hybridization, 0.25 mg of the beads were treated with 40 units of MBN for 20 min at room temperature in 200 μL of the reaction buffer containing 30 mM sodium acetate (pH 5.0), 50 mM sodium chloride and 1 mM zinc chloride^47, 48^. The beads were then washed with 10 mM PBS 3 times. After centrifugation, 200 μL of 50 mM NH_4_HCO_3_ were mixed with the beads and followed by the addition of 1 μg of sequencing grade trypsin and incubation at 37 °C for 24 h. Then, 10 μL of 0.1% TFA were added to stop the reaction. After that, the tryptic mixture, with an addition of 100 μL of the internal standard solution, was transferred into a microspin C18 column (The Nest Group, Inc., MA, USA). The column was preconditioned using 100 μL of ACN and 100 μL of water in advance. After loading, the column was washed with 50 μL of 5% ACN containing 0.1% TFA and eluted with 50 μL of 80% ACN containing 0.1% FA. The elution was repeated for another 3 times. For additional detailed information, please see the manufacturer’s operating instruction.

### LC-MS/MS Method Development and Validation

The LC-MS/MS analysis was performed using an Agilent Series 1290 UPLC system (Agilent Technologies, Waldbronn, Germany) coupled to a 6460 Triple Quad LC-MS mass spectrometer (Agilent Technologies, Santa Clara, USA). Chromatographic separation was achieved using an Agilent SB C18 (2.7 µm, 30 mm × 2.1 mm) at room temperature. The analysis was performed in the positive ion mode. The mobile phase A was 0.02% FA in water, and the mobile phase B was 0.02% FA in methanol with a flow rate of 0.3 mL/min. The elution gradient started with 10% of eluent B run at isocratic conditions for 1 min, and then, it was increased to 90% in 4 min and held for another 4 min. Finally, the initial condition was reached again in 1 min. The sample injection volume was set at 5 μL.

The mass spectrometer was equipped with an electrospray ion source. The following parameters were used: Q1 and Q3: set at unit resolution; the drying gas: 10 L/min, 350 °C; needle voltage: 4000 V; nebulizer pressure: 35 psi. The data were obtained and processed using the Agilent MassHunter Workstation Software (version B.06.00)

The precision and accuracy of the assay were determined using QC samples. The detailed validation procedures and acceptance criteria for the assay have been discussed in a number of studies^31, 49^.

### Method Comparison

The qRT-PCR was conducted for comparison. For the experimental details, please see the Supporting Information.

### Ethical Approval

This study was approved by the Institutional Review Board of Nanjing Medical University, Nanjing, China.

## Results and Discussion

### A Quasi-direct Targeted Proteomics Approach

**Figure 2:**
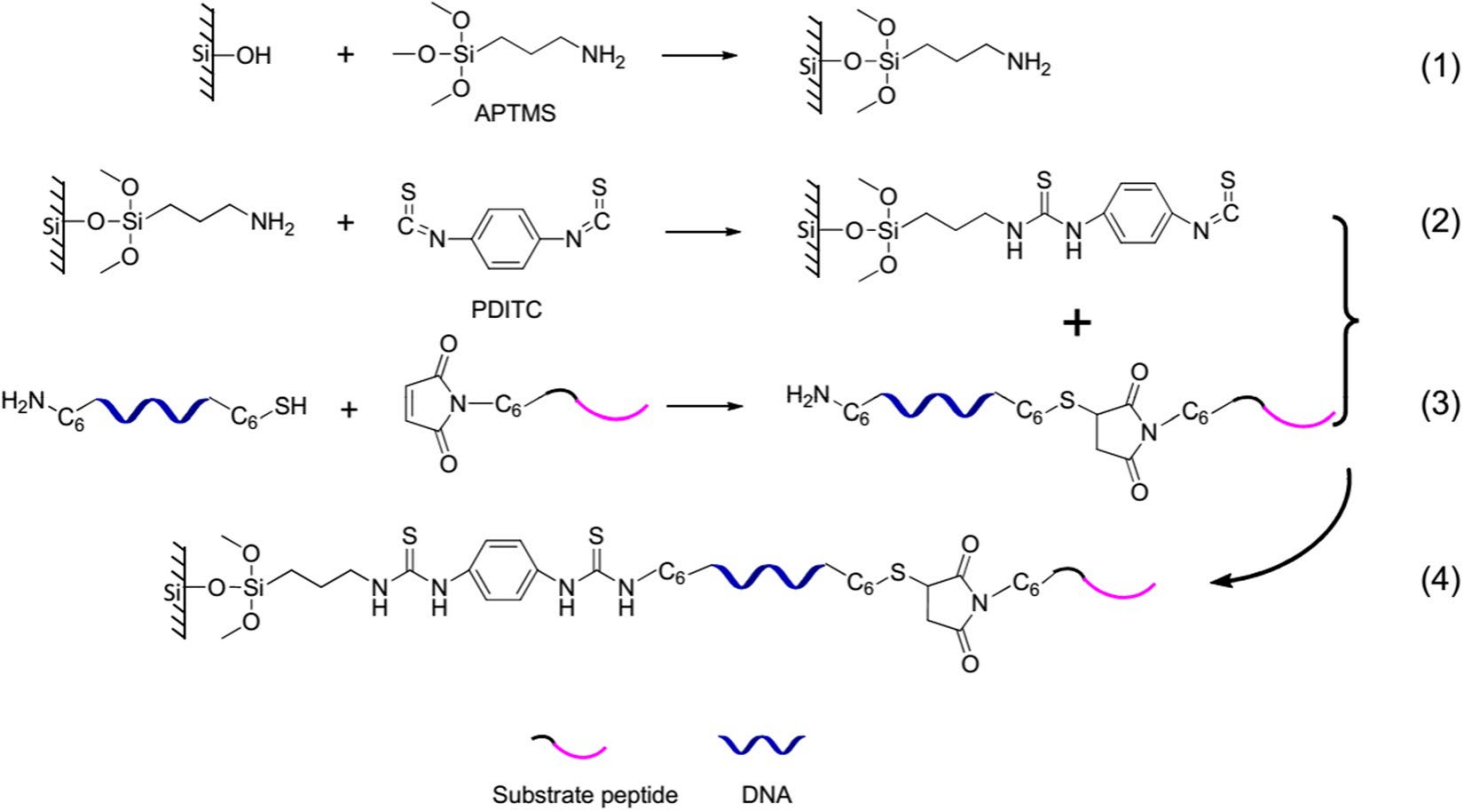
Schematic representation of detailed chemical reactions used to prepare DNA-peptide probe and immobilize the probe on the surface of amino-modified silica beads.

The goal of this approach was to convert the miRNA signal into a reporter peptide for mass spectrometric analysis. For the scheme to work, an appropriate reporter/substrate peptide must be found. Then, the N-terminal maleimidohexanoic acid-modified substrate peptide was conjugated with the 3′-end thiol-modified complementary DNA to form the DNA-peptide probe (Fig. 2, reaction 3). Instead of the previous modification of the target miRNA, the DNA-peptide probe was covalently immobilized on the amino-modified silica nanoparticles *via* a PDITC reaction in this study (Fig. 2, reactions 1, 2 and 4). After reacting with the target miRNA (i.e., miR-21) under the optimized conditions, the miRNA-DNA-peptide hybrids were formed. The excess single-stranded DNA-peptide probe was degraded *via* a single-stranded specific nuclease MBN. Finally, the beads bearing the hybrids were subject to trypsin digestion. Thus, the reporter peptide could be released from the beads and detected using LC-MS/MS.

### Selection of the Reporter and Substrate Peptides

In our previous work, the criteria for the selection of suitable reporter and substrate peptides were extensively described^38^. While evidence indicated that our previously designed surrogate/reporter peptide was applicable to targeted proteomics analysis, these peptides may not be suitable for the present work because of the probe immobilization process.

To date, oligonucleotide immobilization has been widely used in a number of important molecular applications^50^. There are several strategies available for immobilization^51^. Among them, the most accessible approach is covalent attachment of oligonucleotides to the solid support^52^. Normally, oligonucleotides are modified at their end-group and then react with the functional groups on the solid support^53, 54^. The combinations of oligonucleotide/surface functional groups that have been reported so far include thiol/acrylamide^55^, amine/isothiocyanate^52^, activated carboxylic acid/amine^56, 57^, amine/aldehyde^58, 59^ and epoxide/amine^60^. Among them, the amine/isothiocyanate reaction under mildly alkaline conditions can provide a high immobilization efficiency for oligonucleotides, which has been proven by many studies^52, 61, 62^. Thus, PDITC, a member of the isothiocyanate family, was selected to crosslink the DNA-peptide probe to the beads. In principle, this cross-linker is capable of reacting with the amino-modified beads at one end and with the DNA-peptide probe at the other end (Fig. 2, reactions 3 and 4)^52^. Its preferred reactivity with a primary amine is essential for the product yield^63^. However, additional consideration is required in this study because of the potential interference from the peptide component of the probe.

**Figure 3:**
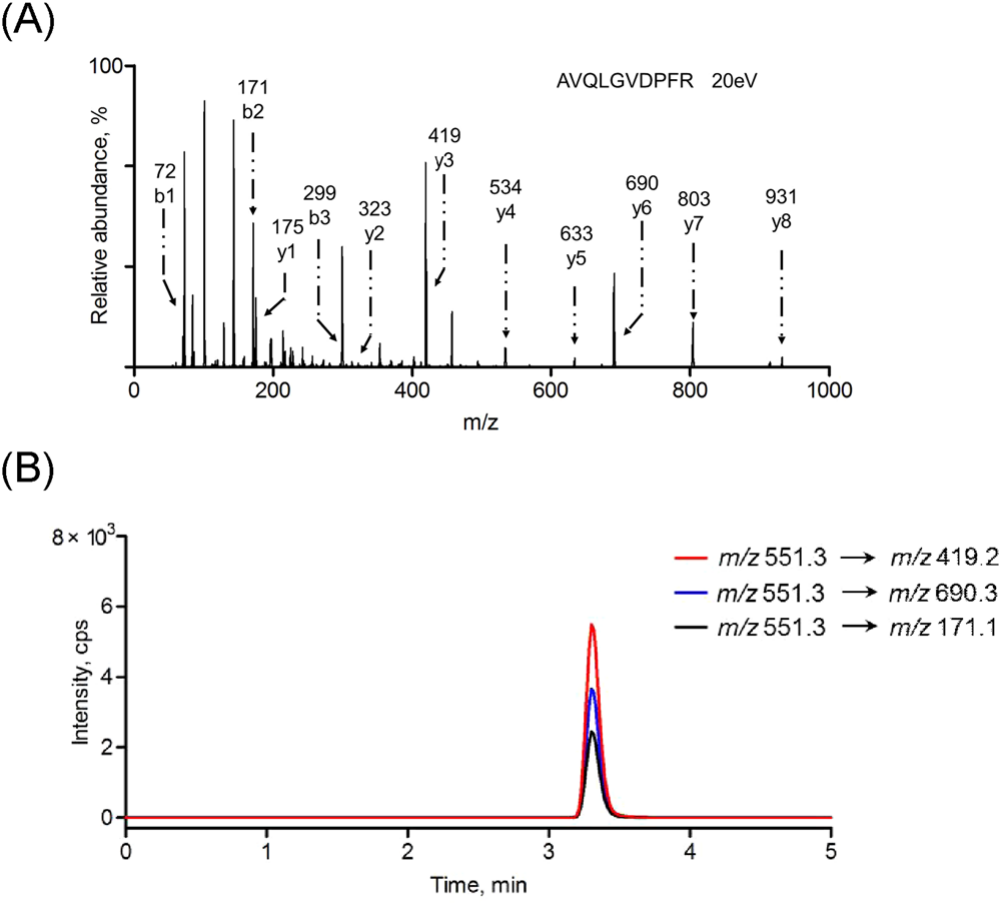
(**A**) Product ion spectrum and (**B**) LC-MS/MS chromatogram of the reporter peptide AVQLGVDPFR. The MRM transitions were *m*/*z* 551.3 → *m*/*z* 690.3, *m*/*z* 551.3 → *m*/*z* 171.1 and *m*/*z* 551.3 → *m*/*z* 419.2.

As is well known, the primary amino groups of the peptides exist both at the N-terminus (α-amino group) and in the side chain of the lysine residues (ε-amino group)^64^. The N-terminal amino group was formerly modified and conjugated with the DNA in the probe formation, whereas the ε-amino group was free and could form a stable phenylthiourea (PTU) derivative during immobilization, if it exists^65^. Thus, the previously selected substrate peptide, GDKAVLGVDPFR, was no longer suitable because of the presence of the lysine residue at position 3. Given the biochemical similarity of lysine and arginine^66, 67^, the replacement of lysine with arginine was easily accomplished while preserving the original tryptic site. The new substrate peptide was GDRAVQLGVDPFR, and the corresponding reporter peptide was AVQLGVDPFR. Fortunately, the sequence of the reporter peptide did not match any protein based on a BLAST search, and the peptide had a high predicted mass response (ESP Predictor (0.90, full score is 1). Experimentally, the maximum response was afforded *via* its doubly charged ion at *m*/*z* 551.3. Its product ion spectrum is shown in Fig. 3A. The sequence-specific b ions and y ions were observed and were indicative of the precursor peptide. The corresponding stable, isotope-labeled peptide was also synthesized to serve as an internal standard. For this peptide, the stable, isotope-labeled [D_8_] Val (V*) was coupled to AVQLGVDPFR at positions 2 and 6 to form AV*QLGV*DPFR, which yielded a molecular mass shift of 16 Da from the non-labeled peptide and a monoisotopic molecular mass of 1117.6 Da. The retention times for AVQLGVDPFR and its isotope-labeled peptide were identical (∼3.3 min, Fig. 1S-A). Finally, the MRM transitions afforded by the product ions, b2 *m*/*z* 171.1, y3 *m*/*z* 419.2 and y6 *m*/*z* 690.3, gave the best signal-to-noise (S/N) and limit of quantification (LOQ) (Fig. 3B). This characteristic pattern was also observed in AV*QLGV*DPFR (Fig. 1S-B). The quantification limit of these MRM transitions was 5 pM (Fig. 2S). Therefore, the peak areas from the three transitions, *m*/*z* 551.3 → *m*/*z* 171.1, *m*/*z* 551.3 → *m*/*z* 419.2 and *m*/*z* 551.3 → *m*/*z* 690.3, were summed and used in the following quantitative analysis^68^.

The digestion efficiency of GDRAVQLGVDPFR was calculated by comparing the response ratios of the equimolar peptide before and after digestion. The estimated value was 99.91% (Fig. 3S). To note, the subsequent conjugated DNA, hybridized miR-21 and the probe-coated silica beads did not have a significant impact on the digestion (please see the following section). The reason may be that the tryptic site was intentionally designed using the three amino acid residues (GDR) and the C_6_ spacer (on DNA) away from the DNA sequence to avoid any potential steric hindrance in the trypsin cleavage. Meanwhile, this design also reduced the potential spatial interference of the peptide tag on the reactivity of the DNA.

### Preparation and Characterization of the DNA-peptide Probe

**Figure 4:**
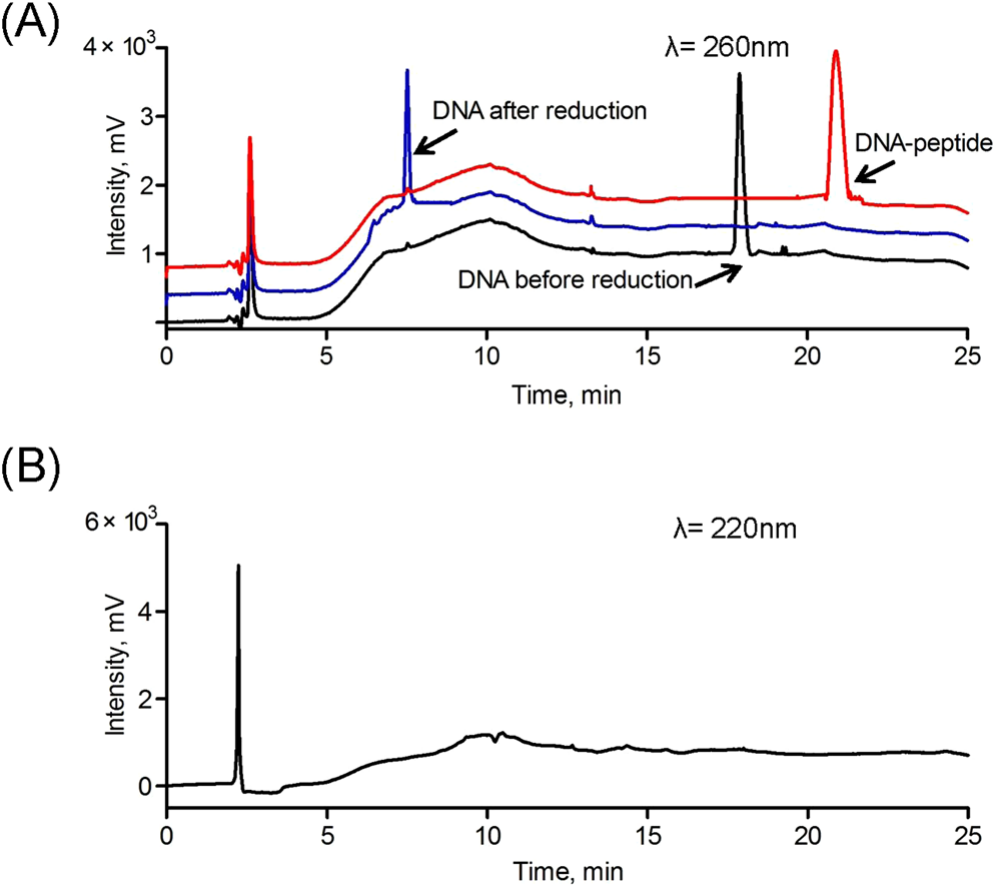
(**A**) HPLC chromatograms of DNA before reduction, DNA after reduction and DNA-peptide conjugation at 260 nm. y-axis scale was adjusted for clarity. (**B**) HPLC chromatogram of DNA-peptide conjugation at 220 nm.

**Figure 5:**
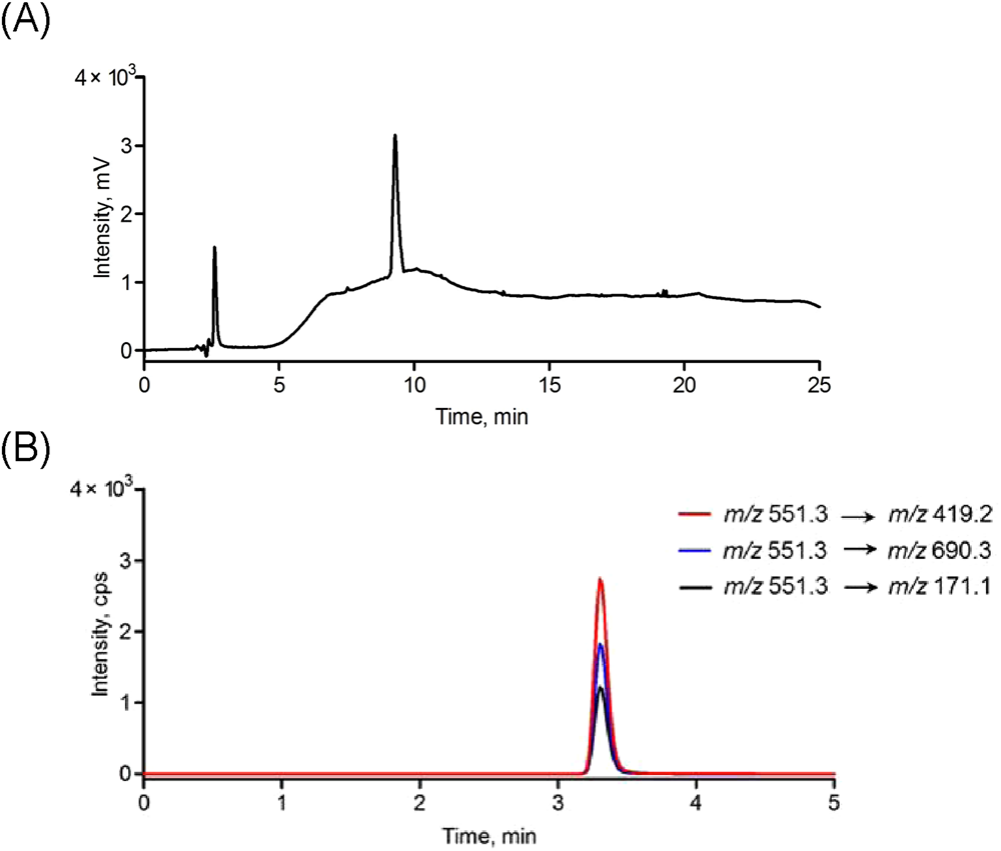
(**A**) HPLC chromatogram (260 nm) and (**B**) LC-MS/MS chromatogram of the trypsin digestion products of DNA-peptide probe.

To prepare the DNA-peptide probe, the disulfide at the 3′ end of the DNA was first reduced to a thiol, and, then, a Michael addition reaction was performed between the reactive thiol and the maleimide at the substrate peptide N-terminus to form the thiol-maleimide linkage (Fig. 2, reaction 3)^69^. In addition to the factors previously discussed^65^, there is another factor that deserves consideration. The newly designed amino-modified 5′ end of the DNA as well as its thiol group at the 3′ end can react with the double bond of the maleimide to form stable carbon-nitrogen and carbon-sulfur bonds, respectively^70^. Fortunately, there is evidence that the maleimide reaction is specific for thiols at pH 6.5 ~ 7.5 and for the amino group when the pH is greater than 8^71^. After careful evaluation of the pH effect, 10 mM PBS (pH = 7.35) was selected as the conjugation buffer in this study. To further rule out the possibility of cross-reactivity (Fig. 4S), we used disulfide-protected DNA to react with the peptide. As shown in Fig. 5S, no new compound was observed in the HPLC chromatogram.

Under the above optimized conditions, almost all the DNA molecules were conjugated with the substrate peptide in the presence of excess peptide. The product was immediately purified using preparative HPLC. As shown in Fig. 4A, the DNA before/after reduction and the DNA-peptide were detected in the HPLC chromatograms using a detection wavelength of 260 nm; their retention times were 7.8 min, 16.8 min and 21.4 min, respectively. The peptide was undetectable at this wavelength. To ensure that the DNA-peptide conjugate we collected did not co-elute with the substrate peptide, a 220 nm detection wavelength was chosen for peptide detection under the same conditions. Figure 4B indicates that the substrate peptide was not eluted out of the column.

For another probe characterization, trypsin was added to the collected fraction. The expected products, DNA-GDR and AVQLGVDPFR (i.e., reporter peptide), were detected using HPLC and LC-MS/MS, respectively. Consistent with the previous observation^38^, the retention time of the DNA product (9.25 min in HPLC) was not the same as that of the premier DNA because of the addition of three amino acid residues from the substrate peptide (Fig. 5A). However, the retention of the peptide product agreed with that of the reporter peptide (Fig. 5B). Furthermore, the estimated amount of the reporter peptide was not significantly different from the amount of the DNA-peptide reactant, which confirmed that the conjugation of the DNA to the substrate peptide did not have a significant impact on digestion.

### Optimization of the DNA-peptide Probe Immobilization

As mentioned earlier, a variety of immobilization strategies, including entrapment, adsorption and chemical binding, have been used to attach oligonucleotide to solid supports, such as ceramics, silicon, glass, magnetic beads, nylon and polymers^57, 72–74^. Among them, covalent immobilization, owning to the involvement of chemical derivatization, is one of the most common approaches. In the present study, the silica beads were first silanized *via* APTMS to generate an active amino group prior to the PDITC reaction.

#### Optimization of the APTMS Concentration

**Figure 6:**
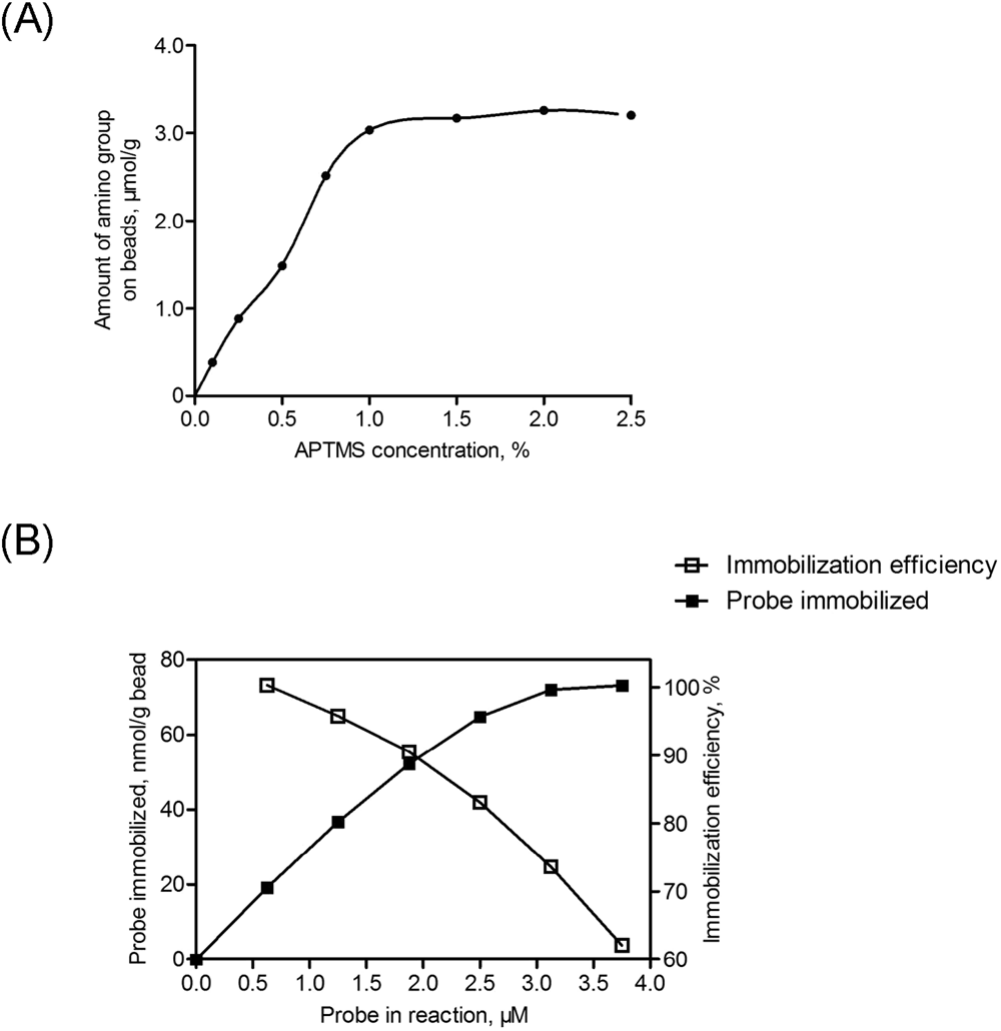
(**A**) APTMS concentration effect on the availability of amino groups. In the analysis of Fmoc, the mobile phase of HPLC was consisted of solvent A (0.025 M potassium dihydrogen phosphate) and solvent B (methanol). Isocratic elution with the mobile phase ratio of A:B (25:75) was employed. (**B**) Relationship between the amounts of total DNA-peptide probe input and immobilized DNA-peptide probe (■). Immobilization efficiency (□) was also determined, correspondingly.

To evaluate the influence of the reactant APTMS concentration on the availability of amino groups on beads, the reaction was performed with an increase in the APTMS concentration from 0.01% to 2.5%. Because it is normally difficult to directly detect immobilized amino groups, a derivatizing agent, Fmoc-Cl, that can react with free amino groups was introduced. After attachment, the Fmoc group was removed, and its absorbance was monitored using a UV detector to estimate the available amino groups^69, 75^. The results indicated that the amount of the amino groups increased as the concentration of APTMS increased from 0.1% to 1% and reached a plateau afterward (Fig. 6A). Therefore, 1% APTMS was used in the subsequent experiments.

#### Optimization of the Probe Concentration

After determining the availability of the amino group, the PDITC reaction was performed with various concentrations of the DNA-peptide probe, ranging from 0 μM to 3.75 μM. The immobilization efficiency was calculated as the input of the DNA-peptide probe divided by the amount of the probe immobilized, which can be estimated from the probe amount left in the solution. As shown in Fig. 6B, there was a direct and positive relationship between the input and the immobilized probe. However, the level of immobilization efficiency decreased throughout the range. This phenomenon may result from the steric hindrance of the immobilized probes that block the access of the free probes to the adjacent amino sites^52^. Taking the immobilization amount and efficiency into account, we chose 1.875 μM as the probe reaction concentration with 87.3% immobilization efficiency.

### Hybridization Efficiency

In this study, the hybridization efficiency was assessed using the molar ratio of the hybridized miRNA and reactant miRNA^76, 77^. Its value was influenced by many parameters, including hybridization buffer, temperature and time. Six commonly used buffers were examined, and a buffer of 10 mM Tris, 100 mM KCl, and 1 mM MgCl_2_ at pH 7.4 (buffer 3) provided the highest efficiency (Table 1S, Fig. 6S). In addition, the optimum temperature and time were 62 °C and 12 h, respectively. Under these conditions, the hybridization efficiency reached 90.2%.

The distinct nature of miRNA concerning detection specificity is the presence of highly homologous family members. Their sequences differ by only 1–3 nucleotides^10, 11^, which may create extra difficulties when it is necessary to distinguish specific miRNAs from their family members. To evaluate the specificity of this assay, we also applied the assay to single-base mismatched miR-21 (SM) and double-base-mismatched miR-21 (DM). The hybridization efficiency decreased in the order of matched (100%) > SM (9.6%) > DM (3.5%). As predicted, the non-complementary sample did not display a peak.

### Mung Bean Nuclease Treatment

**Figure 7:**
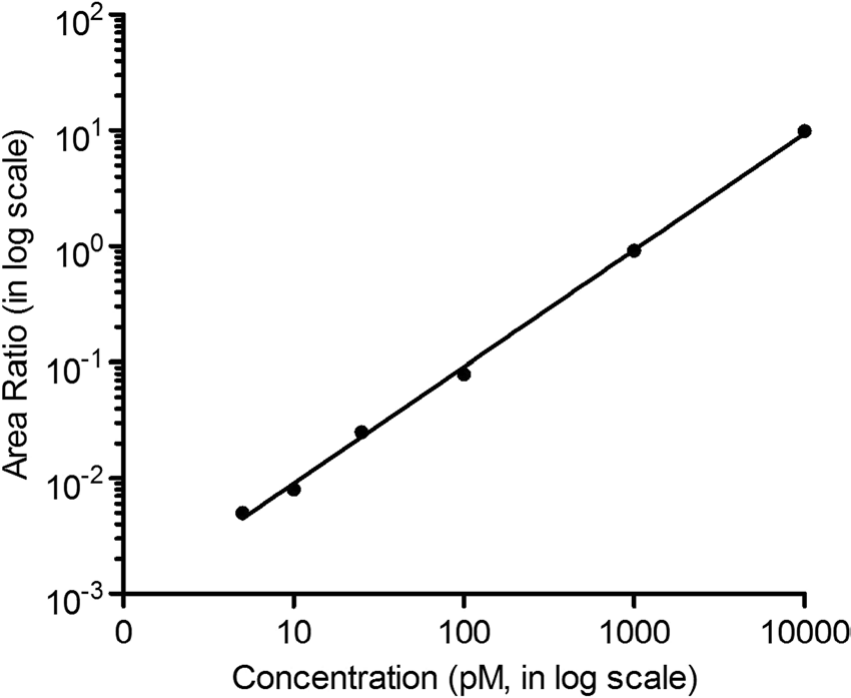
A representative calibration curve (5 pM to 10 nM) for the miR-21 standards. Relative peak area ratio of the reporter peptide and the stable isotope-labeled internal standard with the same sequence was plotted against concentration.

MBN is a single-stranded specific nuclease purified from the sprouts of the mung bean, *Vigna radiate* ^72^. This enzyme prefers to degrade single-stranded DNA (ssDNA) over double-stranded DNA (dsDNA) or DNA-RNA complexed by 30,000-fold^78, 79^. In addition, the enzyme can cleave at a single-nucleotide mismatch when two nucleotide strands are not a perfect match. However, the enzyme can still potentially degrade double-stranded DNA at very high concentrations^78, 80^. Thus, the specificity of MBN was evaluated. The results indicated that up to 200 U/mL of MBN can digest ssDNA completely without affecting dsDNA (Fig. 7S).

### Validation of the Quasi-direct Targeted Proteomics Assay

After determining the conditions for the immobilization, hybridization and digestion, the quasi-direct targeted proteomics assay was validated for further application to biological samples. Calibration curves were generated using a weighted linear regression model with a weighting factor of *1*/*x* ^*2*^. The relative peak area ratio between the reporter peptide and the stable isotope-labeled internal standard was plotted against the concentration. For the assay, the LOQ was 5 pM, and the established concentration range was from 5 pM to 10 nM (Fig. 7). Notably, the LOQ was chosen as the concentration corresponding to the lowest standard of the calibration curve that gave good accuracy and precision as suggested by FDA^81^. However, we have to admit that the LOQ is at least 5 pM in neat buffer conditions, but may be higher when realistic sample matrix is present in the sample. The other results are provided in the Supporting Information (Table 2S).

### Quantification of miR-21 in Breast Cells and Tissue Samples

**Figure 8:**
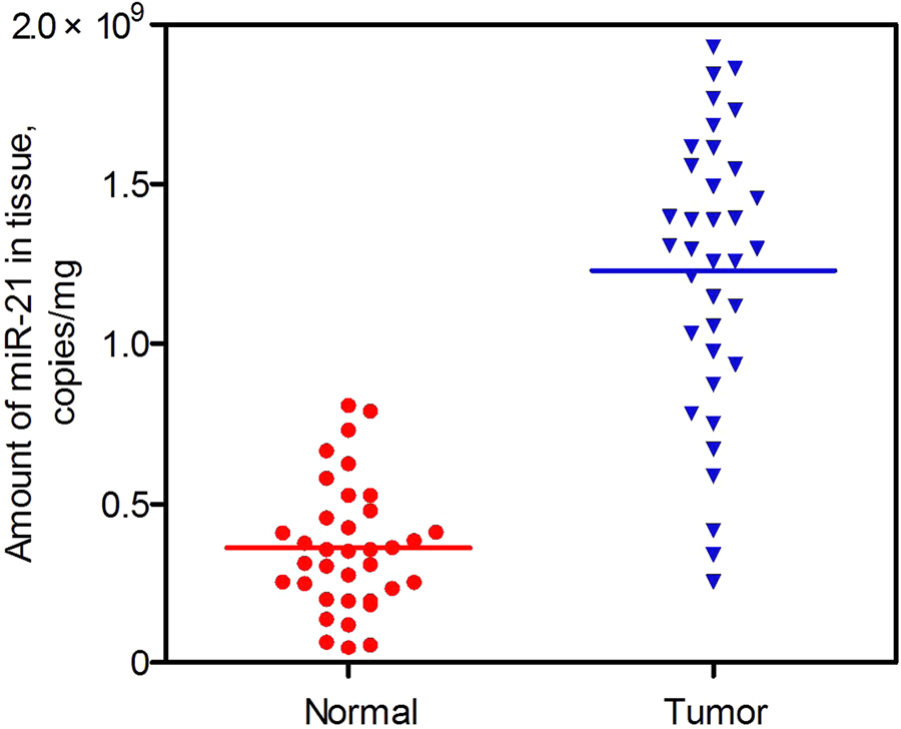
Quantification of miR-21 in 36 matched pairs of breast tissue samples using quasi-direct targeted proteomics assay.

Using the assay, the levels of miR-21 were accurately quantified as (7.94 ± 1.62) × 10^3^ copies/cell in MCF-10A cells, (3.06 ± 0.45) × 10^4^ copies/cell in MCF-7/WT and (4.90 ± 0.66) × 10^4^ copies/cell in MCF-7/ADR cells. Their difference was statistically significant (p < 0.05). This result agrees well with the results reported in our previous work^38^, but all the values are higher. One of the possible reasons could be the indirect nature of the previous method, i.e., miR-21 was biotinylated and bound to streptavidin agarose. The biotinylation process may cause information loss of the target miRNA because of the complexity of the biological samples, e.g., the ubiquitous presence of biotin^44^. Finally, miR-21 was measured using quantitative qRT-PCR for a comparison (Fig. 8S). The level of miR-21 determined using our assay was slightly higher than that obtained using qRT-PCR, but the difference was not significant. Such uncertainty could be attributed to the larger variations in the qRT-PCR experiment. qRT-PCR is the most widely reported method and is regarded as a gold standard for quantifying miRNAs because of its high sensitivity^82^. However, this method suffers from practical issues such as low efficiency, high false positive rates for amplification, and sophisticated and expensive analysis^83^. Currently, most qRT-PCR analyses determine the relative miRNA abundance (often with respect to a non-validated reference miRNA)^84^. Absolute quantification *via* qRT-PCR can provide a quantity of unknowns, but it is labor-intensive^85^. Minor variations in the reaction components, thermal cycling conditions, and mispriming events during the early stages of the reaction can lead to large changes in the overall amount of the amplified product^86^. Furthermore, the subsequent analysis is also mathematically complex^83^. Thus, our assay is able to provide the miR-21 level in copies per cell while avoiding complicated sample manipulation and data analysis.

Moreover, we also performed a quasi-direct targeted proteomics analysis of 36 matched pairs of breast tissue samples. The amounts of miR-21 were (3.63 ± 1.99) × 10^8^ copies/mg (range: (0.49–8.10) × 10^8^ copies/mg) in normal tissues and (1.23 ± 0.44) × 10^9^ copies/mg (range: (0.23–1.93) × 10^9^ copies/mg) in tumors (Fig. 8). A two-way comparison using the Mann-Whitney test showed that the tumor samples had a significantly higher level of miR-21 compared to the normal samples (p < 0.0001). Specifically, approximately a 3.4-fold increase in the concentration of miR-21 was observed in tumors.

## Conclusions

In this study, we immobilized the DNA-peptide probe on amino-modified silica beads and developed a quasi-direct targeted proteomics assay for miRNA quantification. Using this assay, the target miR-21 was quantified in 3 breast cell lines and 36 pairs of breast tissue samples. The advantages of the quasi-direct targeted proteomics approach were demonstrated by converting the miRNA signal to the mass response of the reporter peptide. More importantly, immobilization of the DNA-peptide probe circumvents the miRNA biotinylation and subsequent dependence on the biotin-streptavidin interaction and allows the use of RNA samples without any further manipulation. Technically, this quasi-direct targeted proteomics method can be easily applied to other miRNAs by replacing the DNA sequence with one that is complementary to the target miRNA while keeping the reporter peptide the same. However, the parameters, including conjugation, immobilization, hybridization and digestion, deserve careful optimization to achieve the highest sensitivity and specificity for each miRNA. Furthermore, this assay has more potential for simultaneous detection of multiple miRNAs. Indeed, a key advantage of the LC-MS/MS-based quasi-targeted proteomics assay is its multiplexing ability, which is valid as long as a mass spectrometer can manage the concomitant analysis of multiple reporter peptides while retaining a degree of selectivity. However, the challenges of optimizing the assay format for each peptide, selecting a common dilution factor, addressing the variability and cross-interference, and establishing a robust quality control algorithm are substantial and require further analytical and statistical development. We anticipate that the quasi-targeted proteomics approach described here can ultimately be applied to the profiling of miRNAs in biological samples. This type of quasi-direct analysis may increase the quantitative accuracy and precision, but more evidence is required to confirm this feature.

## Electronic supplementary material

Supplementary Information
