# Supplementary material for: A Quasi-direct LC-MS/MS-based Targeted Proteomics Approach for miRNA Quantification via a Covalently Immobilized DNA-peptide Probe
Source: Sci Rep. 2017 Jul 18;7:5669. doi: 10.1038/s41598-017-05495-7 (PMC5515972; doi:10.1038/s41598-017-05495-7)
Supplement: Supplementary file 1 — Supplementary Information [file 41598_2017_5495_MOESM1_ESM.doc]

**Supporting Information For**

**A Quasi-direct LC-MS/MS-based Targeted Proteomics Approach for miRNA Quantification *via* a Covalently Immobilized DNA-peptide Probe**

*Liang Liu^a^, Qingqing Xu^a^, Shuai Hao^a^, Yun Chen^a^,**

^a^ Nanjing Medical University, School of Pharmacy, Nanjing, 211166, China

***Correspondence**: Dr. Yun Chen, School of Pharmacy, Nanjing Medical University, 818 Tian Yuan East Road, Nanjing, 211166, China

**Table of Contents:**

Table 1S. Hybridization buffers.

Table 2S. Accuracy and precision of LC-MS/MS assay.

Figure 1S. Extracted precursor ion chromatograms of AVQLGVDPFR and AV*QLGV*DPFR, and product ion spectrum of AV*QLGV*DPFR.

Figure 2S. LC-MS/MS chromatograms of the LLOQ and matrix blank.

Figure 3S. LC-MS/MS chromatograms of Male-GDRAVQLGVDPFR and AVQLGVDPFR (A) before and (B) after trypsin digestion.

Figure 4S. Possible side reaction between DNA and substrate peptide.

Figure 5S. HPLC chromatograms of the disulfide-protected DNA before and after reaction with the substrate peptide in PBS.

Figure 6S. Optimization of hybridization buffer (A), temperature (B) and time (C).

Figure 7S. HPLC chromatograms of ssDNA (A) and dsDNA (B) before and after MBN digestion.

Figure 8S. qRT-PCR of miR-21 in MCF-10A, MCF-7/WT and MCF-7/ADR cells.

S1. HPLC Condition for DNA-peptide Probe Purification and Analysis

S2. qRT-PCR

S3. Protocol after Optimization

**Supplementary Tables**

Table 1S. Hybridization buffers.

Table 2S. Accuracy and precision of LC-MS/MS assay.

**Supplementary Figures**

**Figure 1S.** (A) Extracted precursor ion chromatograms of AVQLGVDPFR and isotope-labeled internal standard AV*QLGV*DPFR. (B) Product ion spectrum of AV*QLGV*DPFR.

**Figure 2S.** LC-MS/MS chromatograms of (A) the LLOQ of the reporter peptide AVQLGVDPFR and (B) matrix blank. For the LLOQ, the estimated amount of the reporter peptide on column is ~25 amol.

**Figure 3S.** LC-MS/MS chromatograms of the maleimidohexanoic acid-modified substrate peptide Male-GDRAVQLGVDPFR and the reporter peptide AVQLGVDPFR (A) before and (B) after trypsin digestion.

**Figure 4S.** Possible side reaction between amino-modified DNA and maleimidohexanoic acid-modified substrate peptide.

**Figure 5S.** HPLC chromatograms of the disulfide-protected DNA before and after reaction with the maleimidohexanoic acid-modified substrate peptide in PBS. y-axis scale was adjusted for clarity.

**Figure 6S.** Optimization of hybridization buffer (A), temperature (B) and time (C).

**Figure 7S.** HPLC chromatograms of ssDNA (A) and dsDNA (B) before and after MBN digestion.

**Figure 8S.** qRT-PCR result of miR-21 in MCF-10A, MCF-7/WT and MCF-7/ADR cells. Specifically, the levels of miR-21 were accurately quantified as (7.01 ± 2.06) × 10^3^ copies/cell in MCF-10A cells, (2.20 ± 0.61) × 10^4^ copies/cell in MCF-7/WT cells and (3.94 ± 0.82) × 10^4^ copies/cell in MCF-7/ADR cells.

**Supplementary Experimental Procedures**

S1. HPLC Condition for DNA-peptide Probe Purification and Analysis

The purification of DNA-peptide was performed using a Waters preparative HPLC system consisted of a Waters 2535 solvent delivery pump and a Waters 2489 UV detector (Waters, MA, USA), and an XBridge Prep C18 OBD column (5.0 μm, 19 mm × 150 mm; Waters, USA) at room temperature. The mobile phase was consisted of solvent A (50 mM triethylamine in water, pH = 7.6) and solvent B (ACN). A linear gradient with a flow rate of 10 mL/ min was applied in the following manner: B 5% (0 min) → 16% (5 min) → 20% (20 min) → 5% (25 min) → 20% (25 min). The injection volume was 5 mL. The detection wavelength was set at 260 nm. Fractions were collected.

The collected fraction containing DNA-peptide probe was analyzed using a HPLC system consisted of a Shimadzu LC-20AB solvent delivery pump, a Rheodyne manual valve injector, and a Shimadzu SPD-20A UV-*vis* detector (Shimadzu Corporation, Tokyo, Japan), and a Biobasic HPLC C8 column (5 mm, 4.6 mm × 150 mm; Thermo, USA) at room temperature. The mobile phase and gradient was the same as preparative HPLC. The data was acquired and processed with LabSolutions/LC solution version 1.2.

S2. qRT-PCR

Reverse transcription reaction contained 1μg RNA and 5 nM stem-loop reverse transcription primer (Bulge-Loop^TM^ miRNA primer from Ribobio, Guangzhou, China) in a solution with 0.8 U/μL reverse transcriptase, 4 U/μL RNase inhibitor and 0.2 mM dNTPs in 25 μL volume. The solution was from RevertAid^TM^ First-strand cDNA Synthesis Kit (Thermo Scientific, MA, USA) and used according to manufacturer’s instruction. A calibration curve (10^2^~10^6^ copies of synthetic miR-21) was generated in parallel with biological samples, providing a means to estimate absolute amount of miR-21 in samples. qRT-PCR was performed on 7500 real-time PCR system (Applied Biosystems, CA, USA).20 μL of PCR solution included 2 μL RT product, 10 μL SYBR Green Mix, 0.4 μM forward primers and 0.4 μM reverse primers. Cycling parameters were 95°C for 5 min, followed by 40 cycles at 95°C for 15 s and 60°C for 30 s. All the PCR reactions were run in triplicate, including no-template controls. U6 snRNA served as endogenous control for normalization.

S3. Protocol after Optimization

*Preparation of the DNA-peptide Probe*

1. 1 OD of 300 μL of DNA (4.55 nmol) was first reduced using 300 μL of TCEP reducing beads at 37°C for 2 h with vigorous shaking.
2. After centrifugation at 1000 g for 5 min, the supernatant (450 μL) was collected, and an equal volume of 50 nmol of the maleimidohexanoic acid-modified substrate peptide was added to the solution.
3. The conjugation reaction was performed at 37°C for 4 h with vigorous shaking.
4. The DNA-peptide compound was separated from the excess of the non-conjugated DNA and peptide using high performance liquid chromatography (HPLC). Quantification was performed using an external calibration peak area measurement.
5. The collected fraction was ultra-filtered using an Amicon Ultra 3K device (Merck Millipore, Darmstadt, Germany), and 400 μL (10 nmol/mL) of the DNA-peptide solution were obtained.

*Amino Modification of Silica Beads and Amino Loading Estimation*

1. Silica beads (~50 mg) were washed with ethanol and resuspended in 1 mL of ethanol, followed by an addition of 10 μL (1%) of APTMS and shaken for 1 h at room temperature.
2. The beads were washed 5 times with ethanol and resuspended in 1 mL of methylene chloride followed by an addition of 20 μL (0.12 mmol) of DIEA and sonication for 5 min.
3. To estimate the available amino groups, 25.8 mg (0.1 mmol) of Fmoc-Cl were added into the suspension, and the mixture was shaken for 1 h at room temperature. After centrifugation at 5000 rpm for 5 min, the beads were washed with methylene chloride and ACN 5 times, sequentially.
4. The unreacted amino groups were capped using a mixture of 0.2 M acetic anhydride and 0.2 M DIEA in methylene chloride (1 mL for 50 mg of beads) with shaking overnight at room temperature.
5. The beads were then washed with methylene chloride and ACN 5 times. Finally, the Fmoc protecting group was removed using 1 mL of 20% piperidine in DMF. After shaking for 30 min, the supernatant was collected and analyzed using HPLC.

*Immobilization of the DNA-peptide Probe*

1. Amino-modified beads were suspended in 1 mL of DMF containing 10% pyridine and 0.2% PDITC and shaken for 2 h at room temperature. The beads were then washed with DMF 5 times, ethanol 3 times and methylene chloride 3 times.
2. The (0.75 nmol, 75 μL) DNA-peptide probe and 10 mg of the beads were mixed in 400 μL of the reaction buffer with 2 M sodium chloride and 0.05 M sodium borate buffer at pH 8.5. After shaking overnight at 37°C, the beads were washed with PBS 3 times.

*RNA Isolation and miRNA Hybridization with the DNA-peptide Probe*

1. The tissue samples were first thawed to room temperature and rinsed thoroughly with DEPC water.
2. The total RNA was isolated from cells or approximately 50 mg of the tissue homogenates using TRIzol Reagent, and the RNA concentration was estimated using a nanodrop spectrophotometer (Thermo Fisher Scientific Inc., MA, USA). The RNA extracts were stored at -80°C until further processing.
3. The 1 nM RNA solution (200 μL) in a hybridization buffer (10 mM Tris, 100 mM KCl, 1 mM MgCl_2_, pH 7.4) was mixed with 20 μL of the probe immobilized beads (~0.25 mg), and the reaction was conducted in a 62°C oven for 12 h. After hybridization, the beads were thoroughly washed to remove any unbound miRNAs.

*In-solution MBN Digestion and Tryptic Digestion*

1. The 0.25 mg beads were treated with 40 units of MBN for 20 min at room temperature in 200 μL of the reaction buffer containing 30 mM sodium acetate (pH 5.0), 50 mM sodium chloride and 1 mM zinc chloride. The beads were then washed with 10 mM PBS 3 times.
2. After centrifugation, 190 μL of 50 mM NH_4_HCO_3_ were mixed with the beads followed by an addition of 1 μg of sequencing grade trypsin and incubation at 37°C for 24 h. Then, 10 μL of 0.1% TFA were added to stop the reaction.
3. The internal standard solution (100 μL) was added to the tryptic mixture, and 50 μL of solution were transferred into a microspin C18 column (The Nest Group, Inc., MA, USA). The column was preconditioned using 100 μL ACN and 100 μL water in advance. After loading, the column was washed with 50 μL of 5% ACN containing 0.1% TFA and eluted with 50 μL of 80% ACN containing 0.1% FA. The elution was repeated 3 times.
4. The elute was collected and subjected to LC-MS/MS.
